# Tinnitus emerging in the context of a COVID-19 infection seems not to differ in its characteristics from tinnitus unrelated to COVID-19

**DOI:** 10.3389/fneur.2022.974179

**Published:** 2022-09-09

**Authors:** Ricardo R. Figueiredo, Norma de O. Penido, Andréia A. de Azevedo, Patrícia M. de Oliveira, Adriana G. de Siqueira, Guilherme de M. R. Figueiredo, Winfried Schlee, Berthold Langguth

**Affiliations:** ^1^Faculdade de Medicina, Centro Universitário de Valença, Valença, Brazil; ^2^Departamento de Otorrinolaringologia e Cirurgia de Cabeça e Pescoço, Universidade Federal de São Paulo, São Paulo, Brazil; ^3^Otosul, Otorrinolaringologia Sul-Fluminense, Volta Redonda, Brazil; ^4^Faculdade de Medicina, Universidade Oswaldo Aranha (UNIFOA), Volta Redonda, Brazil; ^5^Department of Psychiatry and Psychotherapy, University of Regensburg, Regensburg, Germany

**Keywords:** tinnitus, hearing loss, COVID-19, SARS-CoV-2, coronavirus infection

## Abstract

**Background and aim:**

COVID-19 is a respiratory disease caused by the new coronavirus SARS-CoV-2, for which the first cases were reported in China, by December 2019. The spectrum of clinical presentations is wide, ranging from asymptomatic cases to a severe acute respiratory syndrome, sometimes with multiple systems involvement. Viral infections, including those related to respiratory virus, may cause hearing loss and, by extent, considering its pathophysiology, tinnitus. A systematic review on inner ear related symptoms in patients with COVID-19 reported 4.5% occurrence rate of tinnitus, with high variance of prevalence between the studies. Our aim is to further explore the relationship between COVID-19 and tinnitus. For this purpose we analyzed a sample of people who had suffered from a COVID-19 infection in the city of Volta Redonda, Brazil. In detail, we compared those with new onset tinnitus during or after the COVID-19 infection with those without tinnitus and those with tinnitus onset before the COVID-19 infection.

**Methods:**

Fifty-seven patients over 18 years old and previously diagnosed with COVID-19 confirmed by a RT-PCR test were included. Patients were subdivided in three groups: no tinnitus (NT), tinnitus that already existed before COVID-19 (chronic tinnitus, CT) and tinnitus that arose during or after COVID-19 (post-COVID-19 tinnitus, PCT). Data concerning COVID-19 symptoms, drugs prescribed for COVID-19, tinnitus characteristics, comorbidities and other otological symptoms were collected. For all the patients, tonal audiometry and otoacoustic emissions were performed. Tinnitus patients fulfilled the Tinnitus Handicap Inventory (THI) and visual-analog scales (VAS) for loudness and distress. Patients with CT answered a simple question about the worsening of their tinnitus after COVID-19.

**Results:**

PCT was reported by 19.3% of the patients, while 22.8% reported CT. No statistical difference was found between CT and PCT concerning hearing function, tinnitus characteristics and tinnitus distress. There was also no statistically significant difference between PCT and NT with respect to COVID-19 symptoms and pharmacological COVID-19 treatment. Patients with CT reported worsening of their tinnitus after COVID-19.

**Conclusion:**

As with other viral infections, inner ear symptoms may be associated with COVID-19. In our sample patients with tinnitus onset before COVID-19 and those with tinnitus onset during or after COVID-19 did not differ significantly in their clinical characteristics and their hearing function, suggesting that tinnitus occurring in the context of a COVID-19 infection is not related to a unique pathophysiological mechanism. The comparison of COVID-19 patients, who developed tinnitus with those who did not develop tinnitus did not reveal any differences in COVID-19 symptoms or COVID-19 treatment. Thus, there was no hint, that a specific expression of COVID-19 is closely related to post COVID-19 tinnitus onset. Although some drugs used to treat tinnitus are known to damage the inner ear cells (especially hydroxychloroquine), we did not see any relationship between the intake of these drugs and tinnitus onset, eventually due to the short prescription time and low doses. Among those patients who had tinnitus before COVID-19 30,8% reported worsening after COVID-19. Overall, tinnitus emerging in the context of a COVID-19 infection seems not to differ from tinnitus unrelated to COVID-19. For further exploring the relationship of tinnitus and COVID-19, large population based studies are warranted.

## Introduction

COVID-19 is a respiratory disease caused by the new coronavirus SARS-CoV-2, for which the first cases were reported in China, by December 2019 (1). The spectrum of clinical presentations is wide, ranging from asymptomatic cases to a severe acute respiratory syndrome, sometimes with multiple systems involvement (2). Until March 15^th^ 2022, 536,590,224 cases, with 6,316,655 deaths were reported worldwide, and 31,611,769 cases, with 668.693 deaths in Brasil (3).

Most frequently reported symptoms include fever, fatigue, myalgia, nasal congestion, rhinorrhea and sore throat (1, 2). Reports of olfactory disorders, including anosmia, hyposmia and dysgeusia, were also frequent, especially with the first waves of infection and probably due to the virus action at the olfactory neuroepithelium, in a similar way to other respiratory tract viral infections (4, 5). In moderate cases, dyspnea may arise, sometimes requiring hospitalization, due to the reduction in blood oxygen saturation (6). More severe cases show severe dyspnea, requiring orotracheal intubation and, sometimes, tracheotomy, and other organs, like the heart, the brain and the kidneys, can be affected (2, 6). In these multiple system cases, other pathophysiological mechanisms, like disseminated intravascular coagulation and immunological mechanisms (the “cytokines storm”) have been implicated (6). Over the course of the COVID-19 pandemic several virus variants emerged, with differences in contagiousness and in its clinical manifestations (7).

Viral infections, including those related to respiratory virus, are known to cause hearing loss and consequently also tinnitus in some cases (8, 9). This association is particularly evident in diseases such as epidemic parotitis and measles, but was also described in herpes zoster, coxackie B infections, acquired immunodeficiency syndrome (AIDS) and influenza (10–12).

The pathophysiology behind hearing loss associated with viral infections involves multiple mechanisms and is not fully understood, as of yet (8). Most of the times, the hearing loss is sensorineural, probably due to direct hair cell damage, patients' inflammatory response, or ototoxicity of drugs used to treat the infections (8). Hearing loss in the context of viral infections is also related to individually increased susceptibility to bacterial and fungal infections (8). The hearing losses range from mild to severe, and may be either temporary or permanent (8, 10). In a recent article, the possibility of subclinical auditory changes in COVID-19 infected patients was suggested, considering the findings of reduction of the otoacoustic emissions in some patients (13).

More recently, a long lasting set of symptoms, the so-called “long COVID-19” syndrome, has been described (14). Among the symptoms attributed to “long-COVID-19” are audio-vestibular symptoms such as hearing loss (7.6%), tinnitus (14.8%), and vertigo (7.2%) (15). Moreover, coronaviruses have been shown to cause peripheral neuropathy (15).

The aim of this article is to compare the characteristics of tinnitus patients who developed tinnitus during or after a COVID-19 infection with those who had tinnitus already before their COVID-19 infection. Furthermore, we compared patients who developed tinnitus after COVID-19 with COVID-19 patients who did not develop tinnitus with respect to their COVID-19 symptoms and treatment.

## Patients and methods

### Study design

Cross-sectional.

### Setting

ENT private clinic. All patients attending the clinic, for any kind of symptom or disease, between November 2020 and November 2021 were asked about a RT-PCR confirmed case of COVID-19, and, when the answer was positive, were invited to participate in the study.

### Inclusion criteria

- Age equal or superior to 18 years old.
- Positive COVID-19 RT-PCR test.

### Exclusion criteria

- Patients not willing or not able to give informed consent.

### Clinical data (assessed by authors 1 and 2)

Clinical data were assessed in interviews with a template questionnaire containing the following information:

- Demographics
- COVID-19 symptoms (fever, fatigue, dysphnea, cough, olfactory symptoms, rhinorrhea, nasal congestion, myalgias),
- COVID-19 treatment
- For patients reporting tinnitus: tinnitus duration, laterality, periodicity, onset, type of sound, associated otological symptoms (dizziness and ear fullness), visual-analog scale for loudness and distress, brazilian portuguese validated version of the Tinnitus Handicap Inventory (16).
- For patients that had tinnitus before the COVID-19 infection, a simple question was answered by the patients: “Did your tinnitus worsen during or after the COVID-19 infection?”.
- General Health: comorbidities (arterial hypertension, diabetes mellitus, dyslipidemia, hypothyroidism, obesity and chronic pulmonar disease), psychiatric disorders (self-reported treatment for anxiety and/or depression).
- Habits: smoking, caffeine consumption.
- Otoscopy and pneumatic otoscopy.

### Audiological data (assessed by authors 3 and 4)

- Pure tone audiometry (Amplaid A177 Plus, TDH 49H headphones, B71 bone conductor).
- Speech testing (Amplaid A177 Plus, TDH 49H headphones).
- Distortion products otoacoustic emissions (DPOAE, Interacoustics Otoread).

For the pure tone audiometry, the criteria of the American Speech-Language. Association were employed (Silman & Silverman for types of audiometric curves and Lloyd & Kaplan for the grade of hearing loss) (17). Frequencies of 250 Hz, 500 hZ, 1,500 Hz, 2,000 Hz, 3,000 Hz, 4,000 Hz, 6,000 Hz, and 8,000 Hz were tested in a sound isolated booth. For speech testing, the speech detection threshold (SRT) was obtained using the phonetic balanced words in Brazilian Portuguese. A percentage of 92% or greater was considered as normal (18). The DPAOE were obtained at 1,500 Hz, 2,000 Hz, 3,000 Hz, 4,000 Hz, 5,000 Hz and 6,000 Hz in a 2*f* 1-*f* 2 ratio, being *f* 1 65 dB, and *f* 2 55dB. The test was considered normal (“pass”) when the signal-to-noise ratio was equal or >3 in ate least four frequencies.

### Statistical analysis

The descriptive analysis of the collected data was presented in tables. Numerical data were expressed by central trend and dispersion measurements, and the categorical data were expressed by frequency (*n*) and percentuals (%).

Patients were subdivided in three groups: no tinnitus (NT), patients who already had tinnitus before COVID-19, lasting for at least 3 months (chronic tinnitus, CT) and patients whose tinnitus arose on the time lapse of up to 1 month after COVID-19 infection confirmation (post-COVID-19 tinnitus, PCT).

- for the analysis of the possible relationship between drugs used by the patients with the intention to treat COVID-19 and tinnitus development, patients were subdivided in two groups, one including the NT patients and the other including the PCT patients.

### Inferential analysis included the following methods

- for the comparison between the three groups (NT, CT and PCT) concerning the numerical data, the Kruskal-Wallis ANOVA (non-parametric) was employed, as well as the Dunn multiple comparisons test (non-parametric). Categorical data were analyzed with the Fisher exact test.

- for comparison between the two tinnitus groups (CT and PCT). The Mann-Whitney (non-parametric) was employed for the numerical data and the Fisher exact test was employed for categorical data. Non-parametrical tests were applied because the variables didn't show normal Gaussian distribution, according to the rejection of the normality hypothesis by the Shapiro-Wilk test and graphical analysis of the histogram.

- The significance level was established at 5% and the statistical analysis was processed with the 26 version of the statistics software SPSS.

### Ethics

The study was approved by the Ethical Committee of the UNIFOA, Fundação Oswaldo Aranha, Volta Redonda, RJ, Brazil (Project Number CAAE 32526620.6.0000.5237). The study was consistant with the Helsinki Declaration for human rights, and all the patients filled in the informed consent after all the study aspects were clarified by the researchers.

## Results

Fifty seven patients were included. Tables 1, 2 show demographic and clinical characteristics of the sample in the different subgroups.

**Table 1:** Numerical variables for the whole sample (*n* = 57).

**Variable**	**NT**					**CT**					**PCT**					***p value***
	***n*/%**	**Median**	**IQI**			***n*/%**	**Median**	**IQI**			***n*/%**	**Median**	**IQI**			
Age (years)	33 / 57.9	45	34	-	60	13 / 22.8	58	39	-	64	11 / 19.3	53	44	-	67	0.12
Tinnitus duratio*n* (months)						13	36	12	-	120	10	1,5	1	-	5.3	**0.0001**
VAS loudness						13	6	4,5	-	6	10	5	3.8	-	7.8	0.97
VAS distress						13	5	2,5	-	8	10	5	3.8	-	10	0.26
THI score						13	28	12	-	47	10	37	11	-	62	0.47

NT, no tinnitus; CT, chronic tinnitus; PCT, post-COVID-19 tinnitus; IQI, interquartile interval; VAS, visual analog scale; THI, Tinnitus Handicap Inventory.

Statistically significant data are indicated in bold.

**Table 2:** Compares COVID-19 symptoms of the NT and PCT groups (Fisher exact test).

**Variable**	**NT**		**PCT**		***p value***
	***n***	**%**	***N***	**%**	
**Fever**					
Yes	16	48.5	7	63.6	0.30
No	17	51.5	4	36.4	
**Cough**					
Yes	21	63.6	7	63.6	0.65
No	12	36.4	4	36.4	
**Dysphnea**					
Yes	7	**21.2**	5	**45.5**	0.12
No	26	78.8	6	54.5	
**Olfactory loss**					
No	8	24.2	2	18.2	0.99
Hyposmia	10	30.3	3	27.3	
Anosmia	15	45.5	6	54.5	
**Parosmia**					
Yes	9	27.3	3	27.3	0.64
No	24	72.7	8	72.7	
**Nasal congestion**					
Yes	13	**39.4**	8	**72.7**	**0.057**
No	20	60.6	3	27.3	
**Rhinorrhea**					
Yes	10	30.3	3	27.3	0.59
No	23	69.7	8	72.7	
**Sore throat**					
Yes	12	36.4	4	36.4	0.63
No	21	63.6	7	63.6	
**Fatigue**					
Yes	18	54.5	8	72.7	0.24
No	15	45.5	3	27.3	
**Myalgias**					
Yes	13	**39.4**	7	**63.6**	**0,15**
No	20	60.6	4	36.4	

NT, No tinnitus; PCT, Post-COVID-19 tinnitus.

Statistically significant data are indicated in bold.

Considering the patients with chronic tinnitus, nine (69.2%) of them reported that their tinnitus didn't get worse after the COVID-19 infection, while four (30.8%) reported worsening.

The comparison between the three groups (NT, CT, and PCT) showed no significant differences concerning gender, ethnics, year of COVID-19 infection (2020 and 2021), otologic associated symptoms, comorbidities, habits and self reported anxiety and/or depression.

When comparing the two groups of tinnitus patients (CT and PCT), no statistically significant diferences were found with respect to tinnitus characteristics such as laterality (bilateral tinnitus most prevalent in CT and PCT groups) and periodicity (constant tinnitus most prevalent in both groups). Concerning tinnitus onset, sudden onset was more prevalent on PCT and gradual onset on CT. The most prevalent type of tinnitus sounds were whistle (30.8%) on CT and wheezing (45.5%) on PCT. Only one case of pulsatile tinnitus was reported, by a patient with chronic tinnitus.

Concerning tinnitus associated symptoms, such as dizziness, headaches and insomnia, no statistically significant differences were found between the CT and PCT groups.

**Figure 1:**
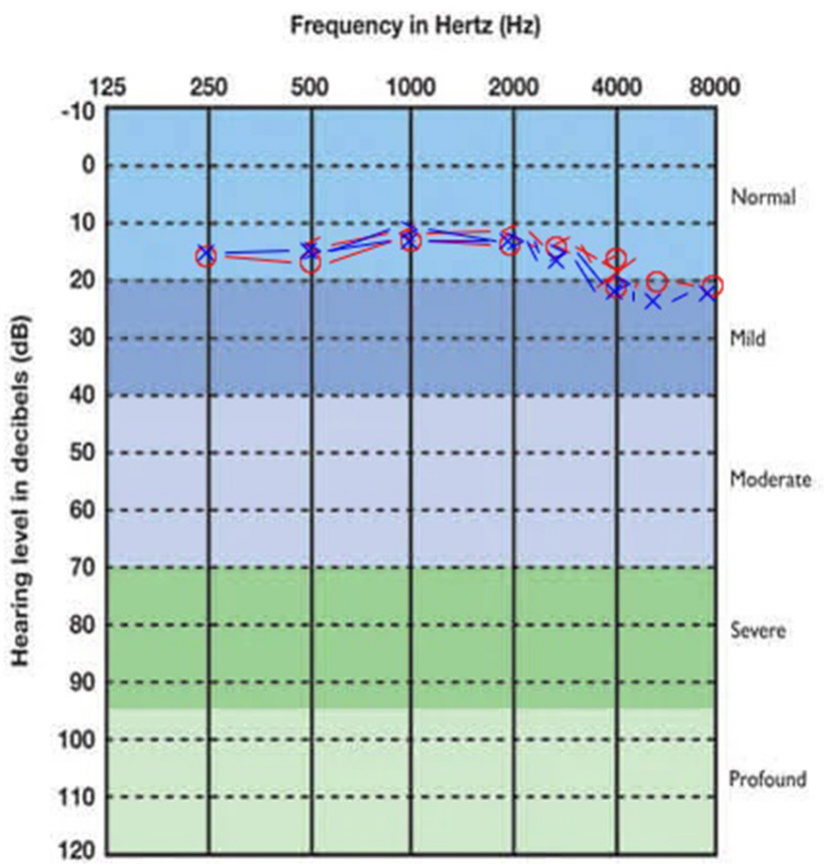
Average audiogram of NT group patients. Right ear thresholds in red, left ear in blue.

**Figure 2:**
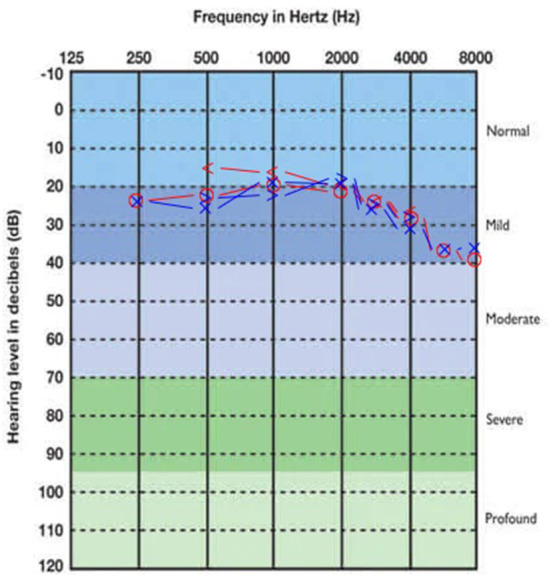
Average audiogram of PCT group patients. Right ear thresholds in red, left ear in blue.

**Figure 3:**
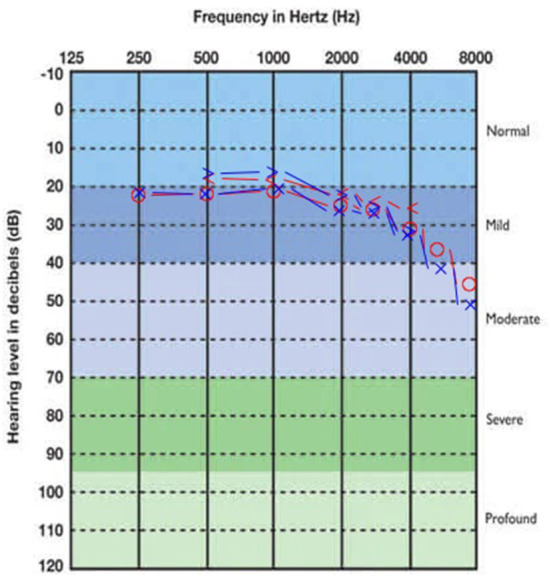
Average audiogram of CT group patients. Right ear thresholds in red, left ear in blue.

Concerning audiometric parameters and otoacoustic emissions, both CT and PCT were associated with hearing loss. Mild sensorineural hearing loss, with a descendant audiogram, was the most frequently found type, both in CT and PCT (Table 3). The SRTs medians were normal in all the three subgroups. Figures 1–3 show, respectively, the average audiograms for NT, PCT, and CT. Ivermectin, azythromicin, nitazoxanide and hydroxychloroquine were, in this order, the most frequently drugs used by the patients with the purpose to treat COVID-19 (Table 4).

**Table 3:** The comparison of otoacoustic emissions findings between the three groups (NT, CT and PCT).

	**NT**		**CT**		**PCT**		
**DPOAE RE**							
Normal	24	75.0	5	38.5	5	45.5	**0.039**
Abnormal	8	25.0	8	61.5	6	54.5	
**DPAOE LE**							
Normal	22	68.8	2	15.4	6	54.5	**0.005**
Abnormal	10	31.3	11	84.6	5	45.5	
Fisher exact test							

NT, no tinnitus; CT, chronic tinnitus; PCT, post-COVID-19 tinnitus; RE, right ear; LE left ear; DPOAE, distortion product otoacoustic emissions.

Statistically significant data are indicated in bold.

**Table 4:** The comparison of their use between the groups.

**Drug**	**NT**		**PCT**		***p value***
	***N***	**%**	***N***	**%**	
**HCQ**					
Yes	6	18.2	2	18.2	0.99
No	27	81.8	9	81.8	
**AZY**					
Yes	16	48.5	7	63.6	0.30
No	17	51.5	4	36.4	
**IVE**					
Yes	22	66.7	9	81.8	0.29
No	11	33.3	2	18.2	
**NIT**					
Yes	10	30.3	5	45.5	0.29
No	23	69.7	6	54.5	

HCQ, hydroxychloroquine; AZY, azythromicin; IVE, ivermectin; NIT, nitazoxanide; NTno tinnitus; PCT, post-COVID-19 tinnitus.

## Discussion

The COVID-19 pandemic hugely affected the entire world. It had not only an impact on the health systems, but also on social and cultural life, economy and psycological well-being. A plethora of acute and chronic, mild and severe, temporary and permanent COVID-19-related symptoms was described, many of them in the otolaryngological domain, and, among those, some audiovestibular symptoms, including tinnitus (19). In this pilot study we aimed to investigate the emergence of tinnitus in the context of a COVID-19 infection.

Considering the prevalence of tinnitus following COVID-19, data are very heterogeneous, ranging from 1.2% (19) to 23.2% (20), and many studies didn't achieve high quality standarts. A meta-analysis published by April 2021 reported an event rate of 4.5% for tinnitus among COVID-19 patients (21). The prevalence of tinnitus emerging in the context of a covid-19 infection was rather high in our sample (19.3% of PCT) (21, 22) presumably due to a selection bias, as study participants were recruited among patients presenting at an ENT clinic. Some authors pointed out that COVID-19-related tinnitus may be a transient phenomenon (19).

In addition to a potential direct effect of the SARS-CoV2 on tinnitus, also COVID-19 related social, cultural and psycological factors may play a role. An online multicenter survey conducted mainly in North America and Europe (23) evaluated the effects of COVID-19 infection on patients who already had tinnitus before COVID-19, and for 40% of them tinnitus got worse, which is partially in line with our findings (30% of worsening). In addition, this study reported that 32% found their tinnitus to be more bothersome due to COVID-19-related fears and restrictions. This is in line with the findings from a longitudinal study, in which patients were investigated before the COVID-19 pandemic and shortly after the beginning of the pandemic (24). At this time tinnitus patients reported more tinnitus complaints, even if they were not affected by a COVID-19 infection (24).

Our data concerning general COVID-19 symptoms are in line with most of the studies (20) that investigated COVID-19 patients during a similar period of data collection. Noticeably, during the course of the pandemic the prevalence of the various virus variants changed, which in turn had an effect on expression of symptoms (7).

Advanced age, comorbidities, such as diabetes mellitus, cardiovascular disease, chronic pulmonar disease, and habits, such as tabagism, have been described as impacting factors in the development of complicated COVID-19 (6, 25). In our study we could not identify any COVID-19 symptoms associated with tinnitus in the context of COVID-19. Our data don't support a potential association between pre-existing depression and post-COVID-19 tinnitus, but these data must be interpreted with extreme caution, as depressive symptoms were not assessed with a validated depression questionnaire. On the other hand a mediating effect of depression on tinnitus development seems plausible (23, 24).

The association between tinnitus and anxiety/depression is well-known (26) and was associated with COVID-19-related tinnitus elsewhere (26). Also, the impact of COVID-19-related distress on tinnitus, including the fears of getting seriously ill and psychological effects of social isolation, has been extensively described elsewhere (23).

Many drugs have been re-purposingly tried to treat COVID-19, in an effort to reduce its morbidity and mortality. Most of them were proved to be innefective or are still being tested, with no proven efficacy, as yet, a fact that has been subject of intense scientific and political debates (27, 28). The most frequently used drugs to treat COVID-19 in Brazil are hydroxychloroquine, azythromicin, ivermectin, and nitazoxanide (28, 29). Of those, hydroxychloroquine, a drug used as anti-malaric and anti-rheumatic, has been linked to ototoxicity, and, subsequently, tinnitus (30). Our data does not show an association between the intake of drugs during the COVID-19 infection and post-COVID-19 tinnitus. However, the lack of an association must be interpreted with caution, considering the small sample size.

As for the evaluation of tinnitus burden of COVID-19 related tinnitus, data are still very scarce. One single study (20) reported a visual-analog scale (VAS) mean score of 5 for patients with post-COVID-19 tinnitus, not specifying if the scale referred to tinnitus loudness or distress. In our sample the mean distress-related VAS was in a similar range, both for chronic and post-COVID-19 tinnitus. Also, we didn't find statistically significant differences between chronic and post-COVID-19 tinnitus patients concerning distress and loudness VAS and THI scores, though THI scores are slightly higher on a descriptive level in post-COVID-19 tinnitus patients (average 37 score, compared to 28 for chronic tinnitus patients). This could be related to the shorter duration of tinnitus in the post-COVID-19 tinnitus group, as it has been shown that THI scores decrease typically with increasing duration of tinnitus (31). Both tinnitus groups fall in the mild impact tinnitus group (32), and the THI scores are in a similar range as samples of previous studies from the same institution (33).

Concerning tinnitus characteristics, according to our data post-COVID-19 tinnitus is very similar to chronic tinnitus, as bilateral and constant tinnitus prevail on both groups. This differs from the data of another study (20), in which intermittent tinnitus was more prevalent on post-COVID-19 tinnitus patients. Sudden onset was more frequent on post-COVID-19 tinnitus in our data, providing a hint for a clearly defined temporal relationship.

There is a direct relationship between tinnitus and hearing loss, and most of the tinnitus patients have abnormal audiometries (26, 34). COVID-19, much like other vírus infections, may cause hearing loss, which has been reported in 3.1% to 8.3% of the general COVID-19 cases (19, 21) and in 13.2% of the post-hospitalization patients (35). Our data show a higher prevalence of hearing loss in both chronic and post-COVID-19 tinnitus patients, which is in line with most of the tinnitus studies (9, 26, 29, 36), although this prevalence may be related to the fact that data was collected in an ENT clinic. The post-COVID-19 tinnitus patients are mostly associated with mild sensorineural descendant curve audiometry, which is also in line with other studies (19, 20, 37). However, we cannot conclude from our data, whether the observed hearing loss was related to the COVID-19 infection or preexisting.

We are well–aware of the weaknesses of this pilot study, which include the small size and a potential selection bias of the sample, the diagnosis of depressive and anxiety symptoms only *via* medical history and the cross-sectional design and which all have to be considered in the interpretation of the data.

## Conclusion

There is a relevant proportion of COVID-19 patients who reported tinnitus onset during or immediately after COVID-19 infection. Post-COVID-19 tinnitus has similar clinical characteristics as chronic tinnitus, which began unrelated from a COVID-19 infection, and is not associated with a particularly greater distress. Neither any specific COVID-19 symptoms nor COVID-19 drug treatment predicted tinnitus development. Further studies, with bigger samples and follow-up of COVID-19-related tinnitus patients are warranted to further elucidate the relationship of COVID-19 and tinnitus.

## Data availability statement

The original contributions presented in the study are included in the article/supplementary material, further inquiries can be directed to the corresponding author.

## Ethics statement

The studies involving human participants were reviewed and approved by Ethical Committee of the UNIFOA, Fundação Oswaldo Aranha, Volta Redonda, RJ, Brazil (Project Number CAAE 32526620.6.0000.5237). The patients/participants provided their written informed consent to participate in this study.

## Author contributions

RF and AA: Study design, data collection, and writing. PO, AS, and GF: Data collection. NP, WS, and BL: Writing. All authors contributed to the article and approved the submitted version.

## Funding

BL and WS received partial funding from the European Union's Horizon 2020 Research and Innovation Programme Grant Agreement (No. 848261).

## Conflict of interest

The authors declare that the research was conducted in the absence of any commercial or financial relationships that could be construed as a potential conflict of interest.

## Publisher's note

All claims expressed in this article are solely those of the authors and do not necessarily represent those of their affiliated organizations, or those of the publisher, the editors and the reviewers. Any product that may be evaluated in this article, or claim that may be made by its manufacturer, is not guaranteed or endorsed by the publisher.
